# Exhaled nitric oxide and urinary EPX levels in infants: a pilot study

**DOI:** 10.1186/1476-7961-9-8

**Published:** 2011-05-16

**Authors:** Fredrik Carlstedt, Dagmara Lazowska, Carl-Gustaf Bornehag, Anna-Carin Olin, Mikael Hasselgren

**Affiliations:** 1 Primary Care Research Centre, County Council of Värmland, Karlstad, Sweden; 2 Public Health Science, Karlstad University, Karlstad, Sweden; 3 Occupational and Environmental Medicine, Institute of Medicine, Sahlgrenska Academy, Gothenburg, Sweden; 4 Department of Public Health and Caring Sciences, Family Medicine and Clinical Epidemiology, Uppsala University, Uppsala, Sweden

**Keywords:** Nitric Oxide, Eosinophil Granule Proteins, Infant, Housing, Allergy and Immunology

## Abstract

**Background:**

Objective markers of early airway inflammation in infants are not established but are of great interest in a scientific setting. Exhaled nitric oxide (FeNO) and urinary eosinophilic protein X (uEPX) are a two such interesting markers.

**Objective:**

To investigate the feasibility of measuring FeNO and uEPX in infants and their mothers and to determine if any relations between these two variables and environmental factors can be seen in a small sample size. This was conducted as a pilot study for the ongoing Swedish Environmental Longitudinal Mother and child Asthma and allergy study (SELMA).

**Methods:**

Consecutive infants between two and six months old and their mothers at children's health care centres were invited, and 110 mother-infant pairs participated. FeNO and uEPX were analysed in both mothers and infants. FeNO was analyzed in the mothers online by the use of the handheld Niox Mino device and in the infants offline from exhaled air sampled during tidal breathing. A 33-question multiple-choice questionnaire that dealt with symptoms of allergic disease, heredity, and housing characteristics was used.

**Results:**

FeNO levels were reduced in infants with a history of upper respiratory symptoms during the previous two weeks (p < 0.002). There was a trend towards higher FeNO levels in infants with windowpane condensation in the home (p < 0.05). There was no association between uEPX in the infants and the other studied variables.

**Conclusion:**

The use of uEPX as a marker of early inflammation was not supported. FeNO levels in infants were associated to windowpane condensation. Measuring FeNO by the present method may be an interesting way of evaluating early airway inflammation. In a major population study, however, the method is difficult to use, for practical reasons.

## Background

Asthma and allergic diseases in children are important public health problems, but they are not fully understood from an aetiological point of view. Allergic diseases usually start in early childhood with food allergies and atopic dermatitis, followed by asthma and rhinitis. These conditions are usually diagnosed in a clinical setting when they are manifest. However, there is a strong need for early and objective markers of preclinical disease, as eosinophilic inflammation, both in clinical and scientific settings.

Foetal environment and early life factors are suggested to programme risk of allergic disease in later life. The study Dampness in Buildings and Health (DBH) showed that asthma and allergies among children are associated with exposure to chemicals such as phthalates from plasticized Poly Vinyl Chloride (PVC), organic compounds associated with cleaning products, and a low ventilation rate in the house [1].

In 1993 it was found that FeNO is elevated in patients with asthma [2]. Since then, measuring FeNO has become a widely used method for evaluating eosinophilic inflammation in the airways among asthmatics [3]. FeNO has also been shown to be raised in infants at increased risk of developing asthma, with a strong correlation to atopic disease and maternal smoking [4,5]. Recently, increased FeNO in one-month-old infants has been shown to predispose to transient early wheeze and recurrent wheeze in the first year of life [6].

Ambient NO mostly affects nasal NO levels, though an association with FeNO has been found in infants [7]. Ongoing or recent airway infections may elevate the FeNO levels, while smoking is known to lower FeNO levels, due to airway epithelial changes [8,9]. Today, there is no standardized method for the measurement of FeNO in infants. Single-breath exhalation, the recommended technique for youths and adults, requires cooperation and is not suitable for infants.

Urinary eosinophilic protein X (uEPX) is a cationic protein, also called eosinophil derived neurotoxin, that is released by eosinophils and can be detected in urine. Urinary EPX levels are known to correlate to eosinophilic inflammation in the lungs, measured by bronchoalveolar eosinophilic cell count [10]. In 3-year-old children, uEPX has been shown to be associated with atopy and allergy-related symptoms [11]. Moreover, it has been shown to predict persistent asthma, and to predict allergic sensitization in children [12]. It shows a circadian rhythm with the highest levels occurring at night, and sampling should therefore be carried out at the same time of day [13].

A positive correlation between FeNO and uEPX has been shown in asthmatic children [14] although more recent data has not confirmed this observation [15]. In children with atopic dermatitis, uEPX levels have been shown to correlate to the severity of disease [16] and have been suggested to be useful for monitoring the progression of allergic disease [11].

Prospective cohort studies with a mother-child design are necessary to better understand the contributing environmental factors. The aim of the present pilot study was to investigate the feasibility of measuring FeNO and uEPX in infants up to six months of age. Furthermore, the study aimed to examine relations between indoor environmental factors and these two biomarkers of inflammation.

## Methods

### General design

The Swedish Environmental Longitudinal Mother and child Asthma and allergy study (SELMA), is an ongoing prospective mother-child study in which exposure to environmental factors during the period of pregnancy and infancy are investigated for their role in the development of asthma and allergies in children (http://www.selmastudy.se). In this type of cohort study of a whole population, it is important to find non-invasive biomarkers for early disease. For practical reasons two different types of bio-markers, one sampled in exhaled air and one in a collected urine sample, were chosen for the present study.

The study was performed at three children's health care centres in Värmland, Sweden. Consecutive infants and their mothers who were scheduled for a regular visit at the age of two or six months were invited to participate with a letter sent to the parents. This included a description of the study, an informed consent form, materials and instructions for urine sampling, and a questionnaire. The mothers were asked 33 multiple-choice questions focusing on symptoms of allergic disease, heredity and housing characteristics. The questionnaire has been validated in an earlier study [1]. This validation showed that PVC flooring is often mistaken for "cork-o-plast" flooring, and that the latter has never, or almost never, been laid in Swedish bedrooms. Data for body weight and body length were collected upon examination and asked for from the mothers.

### Measurement of exhaled NO

FeNO measurements were performed for the mother online with the handheld device NIOX MINO (Aerocrine AB, Stockholm, Sweden) with a detection limit of 5 ppb. This method is in accordance with the American Thoracic Society (ATS) recommendations for online NO measurement [3,17].

For analysis of FeNO-levels in infants, there is no standardized method. In the present study mixed oral/nasal FeNO was measured off-line during tidal breathing with an unseptated face mask (Hans Rudolph Inc) covering the nose and mouth as described by Gabriele et al [18]. Sampling took place during a regular visit at the children's health care centre. Exhaled air was sampled if the unsedated infant succeeded in breathing quietly for at least five breaths into the face mask that was tightly fitted during the whole procedure. No correction for flow was made. Ambient NO was measured before each sampling and no NO-free air was available. FeNO-measurements where ambient NO was > 5 ppb were excluded in order to avoid interactions. Exhaled air was sampled in an inert mylar balloon via a non re-breathing valve. The NO concentration in the exhaled air was measured off-line within 24 hours with the CLD 77 AM nitric oxide chemiluminiscence analyser (ECO-physics GmBH, Dürnten, Switzerland).

### Urinary sampling

Urine from the infant was collected by the parents by means of sanitary towels made of cellulose tissue put into the regular diaper on the night before the visit. The test kit included plastic gloves that were used to squeeze the sanitary towels for the urine, which was subsequently collected in laboratory sampling tubes. All tubes were kept cold and were frozen within 24 hours.

### Measurement of uEPX

Urinary EPX was analysed with an ELISA immunoassay, manufactured by Diagnostics Development, Uppsala, Sweden. The assay was run according to the manufacturer's instructions. In order to minimize the influence of differences in water dilution, uEPX levels were adjusted by the creatinine concentration (uEPX/c).

### Statistical methods

The uEPX/creatinine quotient values were skewly distributed and log-transformed prior to analysis. FeNO levels in both the mothers and the infants were non-normally distributed and the distributions were not corrected by log-transformations. Urinary EPX levels in both the mothers and the infants were non-normally distributed, but corrected by log-transformation.

For normally distributed data, unpaired t-test was used for comparisons between groups. For non-normally distributed data, Mann-Whitney U test was used for differences between groups. Spearman's correlation test was used for correlation analyses. A p-value below 0.05 was considered statistically significant. Values are presented as median, range and interquartile range (IQR). Statistic analysis was performed using SPSS 15.0 for Windows.

The project was approved by the regional ethics committee (Uppsala, Sweden) and written informed consent was obtained from all the participating parents.

## Results

Invitation letters were sent to 209 mother-child pairs, 110 (52%) of which agreed to participate. Of the 110 children in the study, 53 (48%) were girls, 51 (46%) were six months old, and 56 (51%) were two months old.

Twelve of the infants were reported as having a cold at the time of the examination or during the two weeks immediately prior. Eight of the children (7%) were reported as being exposed to tobacco-smoke, either presently or during pregnancy. Furred pets were kept in the homes of 53 (49%) of the infants. Windowpane condensation of any degree was reported in 30% of the homes.

### FeNO levels

**Figure 1:**
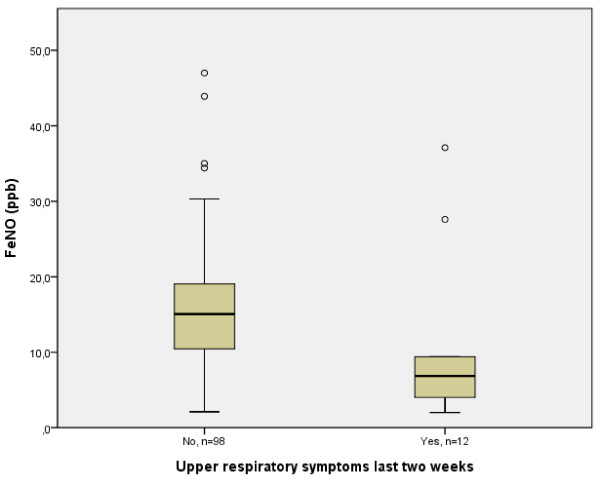
Reported occurrence of reported upper respiratory symptoms in the preceding two weeks and FeNO levels in the infants, circles indicate outliers

FeNO levels in both the mothers and the infants were non-normally distributed and the distribution was not corrected by log-transformation. The FeNO levels were significantly higher in those infants with reported upper respiratory symptoms (URS), such as rhinorrhoea, in the immediately preceding two weeks (p < 0.002, table 1 and figure 1). No such difference was found in the mothers. In the further analyses, FeNO levels of infants and mothers with URS were excluded.

**Table 1:** Median FeNO levels (ppb) in relation to upper respiratory symptoms (URS) in the previous two weeks

	N	Median	Range	IQR
Infants with URS	10	7.0	2-37	5.4
Infants without URS	88	15.0	2-47	8.6
Infants, all	98	14.4	2-47	9.4
Mothers with URS	12	13.0	5-59	9.0
Mothers without URS	92	9.0	5-67	9.0
Mothers, all	104	9.0	5-67	9.0

### FeNO infants, n = 98

The participants were fairly evenly distributed between living in the town centres, suburbs, or countrysides of either Karlstad (90,000 inhabitants) or Filipstad (11,000 inhabitants). About half of the infants were reported to have wooden flooring in their bedrooms. Housing characteristics of the participants are presented in table 2.

**Table 2:** Housing characteristics of 98 infants

	Answer	N (%)
Any smoker in the home	Yes	12(12)
Smoking mother during pregnancy	Yes	5 (5)
Exposure to tobacco smoke during or after pregnancy	Yes	8 (8)
Windowpane condensation, any amount, in the home	Yes	29 (30)
Type of flooring in the infant's bedroom	PVC	27 (28)
	Wood	50 (51)
	Other	21 (21)
Location of the home	Town centre	33 (34)
	Suburb	38 (39)
	Countryside	26 (27)
Size of the home	<75 m^2^	17 (17)
	75-99 m^2^	31 (32)
	>100 m^2^	50 (51)
Type of home	Apartment	42 (43)
	Row house	8 (8)
	Detached house	48 (49)

In all instances ambient NO was low, below 10 ppb. However, at the time of four samplings made on the same day at the same location, ambient NO was found to be more than 5 ppb and these FeNO values were excluded.

No significant correlations were found between FeNO in the infants and weight, weight at birth, age, length, uEPX levels, family size, or FeNO levels of the mothers.

**Figure 2:**
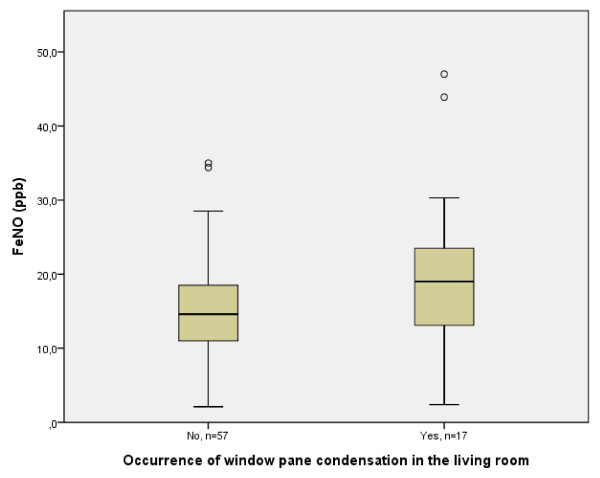
**Reported occurrence of windowpane condensation in the living room and FeNO levels in the infants, circles indicate outliers**.

As seen in table 3, there was a trend towards higher FeNO levels in infants with parent-reported windowpane condensation in the living room (p < 0.05, figure 2), in the parents' bedroom (p = 0.06), and in the infant's bedroom (p = 0.10). Moreover, there was a trend towards lower FeNO-levels in infants from larger homes (p = 0.07, r_2 _= -0.2).

**Table 3:** Median levels of FeNO (ppb) in the infants in relation to parental reported occurrence of window pane condensation

	Yes/no	N	Median	Range	IQR
Living room?	No	57	14.6	2.1-35.0	7.5
	Yes	17	19.0	2.4-47.0	10.4
Parents' bedroom?	No	51	14.6	4.2-35.0	7.5
	Yes	24	18.4	2.1-47.0	10.3
Infant's bedroom?	No	54	14.8	2.1-35.0	7.6
	Yes	19	18.0	2.4-47.0	10.6

There was no significant difference in FeNO levels between different flooring materials in the homes, heredity for allergic disease, tobacco-smoke exposition, reported wheezing, or presence of furred pets in the homes.

### FeNO mothers

FeNO levels were significantly (p < 0.04) higher in subjects living in flats (n 42, median 12, IQR 10) compared to other types of dwelling (n 50, median 8, IQR 7). There was a trend towards lower FeNO-levels in subjects living in larger homes.

There was no significant difference in FeNO levels between mothers with or without asthma, but mothers with reported allergic rhino-conjunctivits (n 24, median 14.0, IQR 14.0) had significantly higher FeNO levels (p < 0.004) than those without (n 68, median 8.0, IQR 9.0). A majority of the measurements were made during the pollen-season. No difference was found in FeNO levels in the mothers related to occurrence of windowpane condensation.

### EPX-levels

There was no difference in EPX levels between infants or mothers with or without reported symptoms of URS during the preceding two weeks (table 4).

**Table 4:** Median levels of uEPX/c (mg/mol creatinine) in the infants and the mothers

	N	Median	Range	IQR
Infants	86	21.4	7.1-81.1	15.5
Mothers	86	20.7	3.6-94.0	16.6

### EPX infants

There was no difference in uEPX/c levels in infants with a history of wheezing, eczema, exposure to tobacco smoke, or atopic heredity. Moreover, there was no significant correlation to body weight, length, or FeNO-levels. EPX levels were significantly higher in infants with reported PVC flooring in their bedrooms. However, when "cork-o-plast" flooring (n = 7) was included in the PVC-group no significance was found.

### EPX mothers

As shown in table 5, the uEPX levels were higher in mothers with self-reported asthma (p = 0.09), allergic rhino-conjunctivitis (p < 0.05) and any allergic disease (p < 0.03). Otherwise no significant differences or correlations with the evaluated parameters were found.

**Table 5:** Median levels of uEPX/c (mg/mol creatinine) in the mothers related to reported allergic disease

	Yes/no	N	Median	Range	IQR
Asthma?	No	76	20.4	3.6-94.0	16.0
	Yes	9	23.4	18.3-57.8	29.0
Rhino-conjunctivitis?	No	59	19.2	3.6-66.1	16.8
	Yes	27	21.9	7.9-94.0	17.1
Any allergic disease?	No	53	16.8	3.6-66.1	16.3
	Yes	32	22.6	7.9-94.0	16.0

## Discussion

The present pilot study investigated the usefulness of two non-invasive methods, FeNO and uEPX measurement, for the evaluation of airway inflammation in infants and their mothers.

The present data support the conclusion that the method used to analyse FeNO in infants may be suitable for a major population study. There was an association between windowpane condensation and elevated levels of FeNO among the infants. Moreover, uEPX was elevated in infants with reported PVC flooring in their bedrooms, and both results may indicate an early proinflammatory effect of indoor air emissions. However, since "cork-o-plast" flooring has never been used in bedrooms in Swedish homes and is often confused with PVC-flooring, this group has to be included in the PVC group, which undermined the relation.

Subjects were recruited among healthy infants in children's health care centres, at a young age. Hence, only a minority has atopic heredity and only a few of the study subjects will develop respiratory disease. This, together with the small sample size, reduces the power of the study.

The method used for measuring FeNO in infants has been criticized for non-accuracy, but in this setting we found a reasonable range and median levels in parity with the mothers. The evident lowering of FeNO levels in those infants with reported upper airway infections is in accordance with what could be expected. This is probably due to less breathing through the nose, making the exhaled air consist of less air affected by the NO-rich nasal cavity. Indeed, it has previously been reported that FeNO levels are reduced in infants with rhinorrhea [19] and URS [4,20]. Despite its large variation, ambient NO has been shown to have little effect on FeNO. The currently recommended technique, which includes inhalation through an NO scrubber, effectively deals with the effect of variable levels of ambient NO on FeNO. In contrast, ambient NO has an effect on measurements of nasal NO when measured with another sampling technique [7]. In the present study nasal NO may influence the FeNO levels due to the sampling technique, and subjects with more than 5 ppb ambient NO at the time of the sampling were therefore excluded from the FeNO analyses. However, when these four subjects were included in the analyses, the reported correlations were strengthened.

The positive correlation between FeNO in the infants and windowpane condensation is in accordance with previous findings among 374 schoolchildren [21]. In that study, those who were non-sensitized to airway allergens and lived in homes with windowpane condensation were found to have higher FeNO levels. Window condensation, as a measure of building dampness, has also been found to be associated with an increase of asthma in preschool children [22].

One could speculate that dampness in buildings could be associated with an altered nasal flow. However, in a study performed among schoolchildren [21], the same association between windowpane condensation and elevated FeNO-levels was found, despite the use of only orally exhaled air.

The trend in the present study that FeNO levels are lower in infants living in larger homes is also seen among the mothers. Living in a larger home is about the same as living in a detached house, and correspondingly lower FeNO levels were seen among mothers living in detached houses, although the same was not the case among the children. Several confounders, such as socio-economic factors and type of ventilation, could be involved in this correlation.

In the cross-sectional DBH study of 10,000 children in Sweden it was shown that reported dampness in the homes is a risk factor for asthma and allergic symptoms among preschool children [23]. The present findings indicate an early influence of dampness in the home on FeNO-levels in infancy. Thus, measuring FeNO by the present method may be an interesting non-invasive way of evaluating early inflammation in the airways. In a major population study, however, the measurement of FeNO would have to be centralized and would therefore not be suitable for a study design using the everyday healthcare system. In addition, it is difficult to use because the balloons must be transported to the laboratory for analysis within 24h. Moreover, 34 children's health care centres are involved in the study and the sampling of the exhaled air is not easily standardized with several performers.

The analysis of uEPX/c in the present study may be questioned due to the sampling method, because this cationic protein adheres to the structures in the sanitary towel. In our own analysis of five healthy volunteers, we found a marked decrease in urinary EPX/c levels after incubation of urine in the sanitary towel. However, this systematic decrease may be of less relevance in a major setting. No normal values are established, but the uEPX/c levels were found to be of about the same magnitude in both the mothers and the children although the sampling method differed between these groups.

No association was found between uEPX/c levels and FeNO, as has been found in previous studies of asthmatic children [24]. The present study subjects are probably too young to have developed any eosinophilic inflammation in the airways. The association in the mothers between allergic symptoms and uEPX/c levels are in accordance with previous findings.

## Conclusions

In the present setting, samples were taken during the first six months of life and few reports of wheezing and allergic symptoms occurred. Urinary EPX may be useful as a non-invasive early marker of inflammation, but in this study and with this sampling method, nothing supports the use of urinary EPX in a major study such as SELMA. FeNO levels in infants were associated to building dampness, measured as windowpane condensation. Measuring FeNO by the present method may be an interesting non-invasive way of evaluating early inflammation in the airways.

## Competing interests

The authors declare that they have no competing interests.

## Authors' contributions

FC carried out the planning of the study, participated in the sampling procedure, performed the statistical analyses and drafted the manuscript. DL carried out most of the sampling and was involved in the planning of the study. CB participated in the planning and analysis of the study and helped to draft the manuscript. AO contributed in the NO-measurements and helped to draft the manuscript. MH carried out the planning of the study, participated in the sampling procedure, the coordination of the study and helped to draft the manuscript.

All authors have read and approved the final manuscript.
